# Pelvic compensation accompanying spinal malalignment and back pain-related factors in a general population: the Wakayama spine study

**DOI:** 10.1038/s41598-023-39044-2

**Published:** 2023-07-22

**Authors:** Shizumasa Murata, Hiroshi Hashizume, Shunji Tsutsui, Hiroyuki Oka, Masatoshi Teraguchi, Yuyu Ishomoto, Keiji Nagata, Masanari Takami, Hiroshi Iwasaki, Akihito Minamide, Yukihiro Nakagawa, Sakae Tanaka, Noriko Yoshimura, Munehito Yoshida, Hiroshi Yamada

**Affiliations:** 1 Department of Orthopaedic Surgery, Wakayama Medical University, 811-1 Kimiidera, Wakayama City, Wakayama 641-8510 Japan; 2 Division of Musculoskeletal AI System Development, Graduate School of Medicine, The University of Tokyo, Bunkyo-Ku, Tokyo, Japan; 3 Spine Center, Dokkyo Medical University Nikko Medical Center, 632 Takatoku, Nikko City, Tochigi Japan; 4 Spine Care Center, Wakayama Medical University Kihoku Hospital, 219 Myoji, Katsuragi-cho, Ito-gun, Wakayama, Japan; 5 Department of Orthopaedic Surgery, The University of Tokyo, Bunkyo-Ku, Tokyo, Japan; 6 Department of Preventive Medicine for Locomotive Organ Disorders, 22nd Century Medical and Research Center, The University of Tokyo, Bunkyoku, Tokyo, Japan; 7 Department of Orthopedic Surgery, Sumiya Orthopaedic Hospital, 337 Yoshida, Wakayama, Japan

**Keywords:** Medical research, Risk factors

## Abstract

Some older adults with spinal deformity maintain standing posture via pelvic compensation when their center of gravity moves forward. Therefore, evaluations of global alignment should include both pelvic tilt (PT) and seventh cervical vertebra-sagittal vertical axis (C7-SVA). Here, we evaluate standing postures of older adults using C7-SVA with PT and investigate factors related to postural abnormality. This cross-sectional study used an established population-based cohort in Japan wherein 1121 participants underwent sagittal whole-spine radiography in a standing position and bioelectrical impedance analysis for muscle mass measurements. Presence of low back pain (LBP), visual analog scale (VAS) of LBP, and LBP-related disability (Oswestry Disability Index [ODI]) were evaluated. Based on the PT and C7-SVA, the participants were divided into four groups: normal, compensated, non-compensated, and decompensated. We defined the latter three categories as “malalignment” and examined group characteristics and factors. There were significant differences in ODI%, VAS and prevalence of LBP, and sarcopenia among the four groups, although these were non-significant between non-compensated and decompensated groups on stratified analysis. Moreover, the decompensated group was significantly associated with sarcopenia. Individuals with pelvic compensation are at increased risk for LBP and related disorders even with the C7-SVA maintained within normal range.

## Introduction

With the increasing aging population, the incidence of adult spinal deformity (ASD) has increased^1^. There has been growing interest in conservative and surgical treatment for ASD that is aimed at extending healthy life expectancy^2–4^. There are several classification systems, and diagnoses are determined by radiographic evaluation of coronal and sagittal inconsistencies^5,6^. In recent years, there has been extensive research on sagittal inconsistencies, quality of life (QoL) scores, and clinical outcomes related to patient health. Sagittal inconsistencies have been demonstrated to be strongly associated with poor QoL^7,8^.

Studies have revealed that evaluation of sagittal alignment is essential in assessing spinal pathology^9,10^. Some older adults maintain the standing position while preventing the center of gravity from moving forward using pelvic compensation. According to Dubousset’s “cone of economy”^11^, a person who is significantly deviated from the center of the sagittal cone needs more energy to maintain the standing position. This sagittal shift and resulting compensatory mechanism generally begin with the loss of lumbar lordosis, which can involve the entire lower extremity and occur on a continuum^11–13^. Meanwhile, compensation restores alignment and enables an upright gait, which helps to maintain and normalize gait patterns that can be disrupted by spinal deformity^12^. As it uses the natural mobility of the hip joint, pelvic compensation is the first compensatory mechanism to restore sagittal balance, which leads to increased pelvic tilt (PT)^13–15^. Therefore, it is insufficient to evaluate global alignment with only the seventh cervical vertebra-sagittal vertical axis (C7-SVA); it is equally important to evaluate pelvic compensation based on PT. Nonetheless, despite its proven clinical value, data on the assessment and characterization of compensatory mechanisms within a large general population are limited. Hence, this study focused on the combination of global sagittal alignment and pelvic compensation to evaluate the standing posture in elderly patients and investigated the factors related to abnormal posturing.

## Results

### Participants’ demographic data and characteristics of the four groups according to the C7-SVA and PT

**Figure 1:**
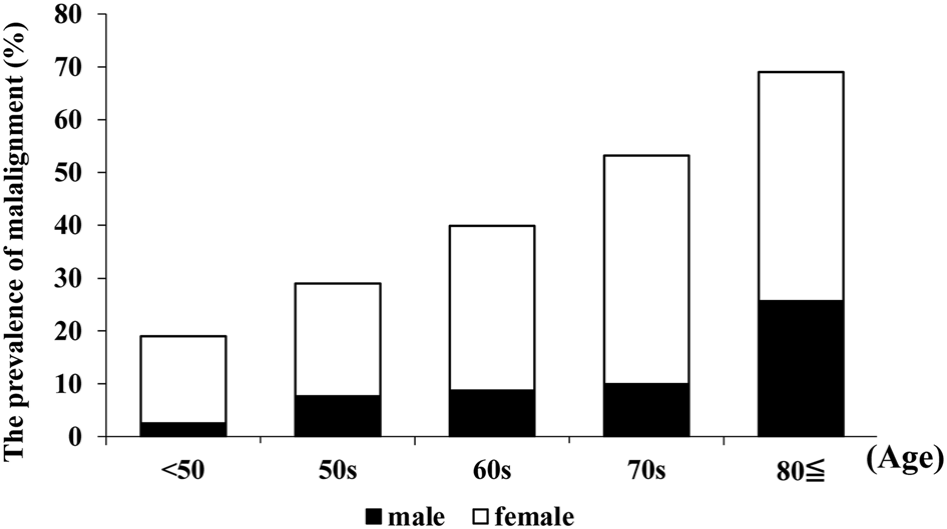
The prevalence of malalignment. The prevalence of malalignment increased with age group: < 50 years, 19%; 50 s, 29%; 60 s, 40%; 70 s, 54%; and > 80 years, 69%. In women, the prevalence of malalignment was significantly higher among those aged > 60 years (40.3% in men vs. 57.6% in women, *P* < 0.0001).

The characteristics of the participants are shown in Table 1. The body mass index (BMI) was significantly lower in women than in men (*P* = 0.027). Table 2 shows the characteristics of the four groups according to the C7-SVA and PT. The number of participants in each group and the average age per group were 606 and 64.3 years in the normal group, 333 and 68.9 years in the compensated group, 53 and 75.0 years in the non-compensated group, and 129 and 76.8 years in the decompensated group, respectively. The proportion of men was 38% in the normal group, 21% in the compensated group, 57% in the non-compensated group, and 20% in the decompensated group. Among the 1121 qualified participants from the Wakayama Spine Study, 515 (126 men and 389 women) had malalignment. The prevalence of malalignment increased with age: < 50 years, 19%; 50 s, 29%; 60 s, 40%; 70 s, 54%; and > 80 years, 69%. In women, the prevalence of malalignment was significantly higher among those aged > 60 years (40.3% in men vs. 57.6% in women, *P* < 0.0001) (Fig. 1).

**Table 1:** Participants’ demographic data.

	Total	Men	Women
Number of participants	1121	355	766
Age strata (years)			
≤ 49	78	26	52
50–59	188	56	132
60–69	319	91	228
70–79	351	105	246
80 ≤	185	77	108
Demographic characteristics			
Age (years)	67.6 ± 11.7	68.5 ± 12.5	67.3 ± 11.4
Height (cm)	155.3 ± 9.1	164.1 ± 7.3	151.2 ± 6.7
Weight (kg)	55.7 ± 10.9	63.1 ± 11.2	52.3 ± 8.9
BMI (kg/m^2^)	23.0 ± 3.5	23.4 ± 3.3	22.9 ± 3.5

**Table 2:** Characteristics of the four groups defined by sagittal vertical axis and pelvic tilt.

	Normal group	Compensated group	Non-compensated group	Decompensated group
Definition	C7-SVA < 50 mm, PT < 20°	C7-SVA < 50 mm, PT ≥ 20°	C7-SVA ≥ 50 mm, PT < 20°	C7-SVA ≥ 50 mm, PT ≥ 20°
Number	606	333	53	129
Age (mean ± SD), years	64.3 ± 11.7	68.9 ± 10.7	75.0 ± 10.4	76.8 ± 7.4
Sex (male:female)	229:377	70:263	30:23	26:103
Body mass index (kg/m^2^)	22.9 ± 3.4	23.1 ± 3.4	23.8 ± 4.0	23.0 ± 3.5
Presence of LBP (%)	33%	43%	44%	57%
VAS of LBP (mm)	10.8 ± 19.2	15.6 ± 23.5	17.8 ± 26.2	24.6 ± 28.1
ODI%	9.1 ± 10.8	13.0 ± 13.3	19.9 ± 17.1	23.3 ± 15.1
Presence of sarcopenia (%)	3.6%	6.6%	17.0%	21.7%

*C7-SVA* Seventh cervical vertebra-sagittal vertical axis; *PT* Pelvic tilt; *SD* Standard deviation; *LBP* Low back pain; *VAS* Visual analog scale; *ODI* Oswestry disability index.

### Prevalence of low back pain and sarcopenia

There was a significant difference in the prevalence of low back pain (LBP), visual analog scale (VAS) score, Oswestry Disability Index (ODI) %, and prevalence of sarcopenia among the four groups (all *P* < 0.0001). Meanwhile, these parameters were not significantly different between the non-compensated and decompensated groups in the stratified analysis (*P* = 0.119, *P* = 0.135, *P* = 0.184, and *P* = 0.472, respectively).

### Factors associated with low back pain and low back pain-related disability

Table 3 shows the factors associated with LBP and related disorders. On multivariate logistic regression analysis, increasing age and BMI were significantly associated with LBP-related disability (ODI% ≥ 21) and sarcopenia, respectively. The compensated and decompensated conditions were significantly associated with LBP (odds ratios [ORs] 1.54 and 2.78, respectively). Malalignment (compensated, non-compensated, and decompensated conditions) was also significantly associated with LBP-associated disability (ODI% ≥ 21) (ORs 1.56, 2.76, and 3.97, respectively). In addition, the decompensated condition was significantly associated with sarcopenia (OR 2.84).

**Table 3:** Factors associated with low back pain and related disability.

	Presence of LBP		ODI% ≥ 21*		Sarcopenia	
	Odds ratio (95% CI)	*p* value	Odds ratio (95% CI)	*p* value	Odds ratio (95% CI)	*p* value
Age (+ 1 years)	1.00 (1.00–1.01)	0.490	**1.05 (1.03–1.07)**	** < 0.0001**	**1.19 (1.14–1.25)**	** < 0.0001**
Sex (female)	1.13 (0.86–1.49)	0.374	1.16 (0.83–1.63)	0.393	1.02 (0.56–1.85)	0.947
BMI (+ 1 kg/m^2^)	1.03 (0.97–1.07)	0.078	**1.12 (1.07–1.17)**	** < 0.0001**	**0.75 (0.69–0.84)**	** < 0.0001**
C7-SVA and PT groups						
Normal	1		1		1	
Compensated	**1.54 (1.16–2.05)**	**0.003**	**1.56 (1.09–2.24)**	**0.016**	1.39 (0.69–2.78)	0.352
Non-compensated	1.68 (0.93–3.02)	0.086	**2.76 (1.47–5.19)**	**0.002**	2.03 (0.72–5.72)	0.181
Decompensated	**2.78 (1.83–4.23)**	** < 0.0001**	**3.97 (2.52–6.24)**	** < 0.0001**	**2.84 (1.39–5.83)**	**0.004**

Significant values (p<0.05) are in bold.

All three statistical models were multiple logistic regression analysis; an LBP-related disability was defined as an ODI% ≥ 21.

Abbreviations: *C7-SVA* Seventh cervical vertebra-sagittal vertical axis; *PT* Pelvic tilt; *CI* Confidence interval; *BMI* Body mass index; *LBP* Low back pain; *ODI* Oswestry disability index.

## Discussion

To the best of our knowledge, this observational study is the first population-based study to focus on the combination of global alignment and pelvic compensation to evaluate the standing posture in elderly patients and investigate factors related to abnormal posturing. We found that the pelvic compensatory function for abnormal posturing changes with age. Moreover, the results suggest that this compensatory effect has sex-specific differences. Groups with pelvic retroversion comprised more women regardless of whether the C7-SVA was maintained within 50 mm; this difference may be owing to sex-specific differences in spinal flexibility and muscle strength^16^. Furthermore, the results suggest that individuals with pelvic compensation are at increased risk of developing LBP and related disorders even if the C7-SVA is maintained within the normal range.

As the aging population continues to grow, so does the prevalence of musculoskeletal diseases, such as spinal disorders. ASD has gained significant attention in the last 10 years, leading to improvements in diagnostic tools, classification schemes, and surgical techniques^2–5^. Furthermore, sagittal malalignment correlates with poor clinical outcomes, LBP, and low health-related QoL in patients with lumbar degenerative disorders, especially those with ASD^6,7^. Previous studies have demonstrated that surgical treatment is superior to conservative treatment in improving outcomes of patients with ASD^14,17^. It is likely that surgical treatment of a grossly disrupted spinal alignment is appropriate^11,12^. We believe that studies that reject conservative treatment for ASD lack detailed consideration of pelvic compensation. Our study revealed that it is insufficient to only treat sagittal inconsistencies and that pelvic compensation must also be considered.

Even if the global alignment is maintained within the normal range, patients with pelvic compensation will experience back pain and decreased performance of activities of daily living. In other words, those who manage to maintain their global alignment within the normal range through pelvic compensation need treatment before the onset of LBP. Kamata et al.^18^ reported that even if the global sagittal alignment is maintained by pelvic compensation in the resting position, pelvic compensation disappears at the start of walking, and global sagittal alignment collapses. Irreversible deformation may occur in the vertebral bodies and intervertebral discs in patients who have undergone failed C7-SVA alignment^19,20^. Individuals for whom the C7-SVA is maintained by pelvic compensation may have LBP due to disuse of the back muscles rather than degeneration of the intervertebral discs or osteoporotic deformation of the vertebral bodies^21^. LBP, as experienced in the compensated group, may be relieved by physiotherapy of the back muscles. Regarding conservative therapies, Takahashi et al.^22^ reported that orthotic treatment (which limits anteflexion so as not to affect back muscles), stretching of the hip extensor muscles, and muscle strengthening are effective. Other studies have reported a high pelvic incidence (PI) as a characteristic of patients who respond to conservative treatment^43^. Specific exercises, such as strengthening of the gluteal muscles, were reportedly effective^44^. The results of the present study clarified that women aged ≥ 60 years with compensated spinal alignment should be considered for conservative therapies. Women aged ≥ 60 years in the compensated group, especially those with a high PI, may benefit from gluteal muscle strengthening. By understanding the appropriate target group for conservative intervention, it is possible to reduce the number of patients requiring surgery for sarcopenia in the decompensated group.

This study has several limitations, including selection bias and a cross-sectional design. As such, a causal relationship cannot be determined, although it is possible to speculate this. Cause-and-effect may be clarified through longitudinal analysis in the future. Compared to the normal group, the compensated group had a higher mean age, and the decompensated group had an even higher mean age, suggesting that the compensated group eventually becomes the decompensated group over time. We speculate that there are two pathways or pathological stages in the progression of adult spinal deformity: the “Normal group → Compensated group → Decompensated group” pathway and the “Normal group → Non-compensated group” pathway. A longitudinal study is planned in the future to clarify the process of progression of exacerbation with age. Another limitation was the exclusion of 341 participants with missing ODI% data from the analysis; we did not perform missing data analysis for these 341 participants since we considered them to be completely random.

## Conclusions

In conclusion, pelvic compensatory function for abnormal posture changes with age, and this compensatory effect differs between men and women. Regardless of whether the C7-SVA was maintained within 50 mm, more women had pelvic retroversion. Furthermore, the results of this study suggest that individuals with pelvic compensation are at increased risk of developing LBP and related disorders even if the C7-SVA is maintained within the normal range.

## Materials and methods

### Participants

This cross-sectional study was approved by the Research Ethics Committees of Wakayama Medical University (no.373) and the University of Tokyo (nos.1264 and 1326). All participants provided written informed consent for their participation and for the publication of the study in print and electronic form. The procedures followed were in accordance with the ethical standards of the responsible committee on human experimentation (institutional and national) and with the Helsinki Declaration of 1975, as revised in 2000. This study assessed a sub-cohort drawn from the Research on Osteoarthritis/Osteoporosis Against Disability (ROAD) study—a large-scale prospective study of bone and joint diseases using population-based cohorts in Japan. The ROAD study, which commenced in 2005, is a nationwide prospective study designed to establish epidemiological indices for evaluating bone and joint diseases. It comprises population-based cohorts from three communities in Japan: a mountainous region in Hidakagawa, Wakayama; a coastal region in Taiji, Wakayama; and an urban region in Itabashi, Tokyo. Details of the study have been described previously^23,24^; therefore, only a brief summary is provided here.

Briefly, subjects included participants of the third visit of the ROAD study, which commenced in 2012 and was completed in 2013. In addition to the former participants, inhabitants of mountainous and coastal areas in the Wakayama Prefecture who were willing to participate in the ROAD survey were also included in the third visit.

**Figure 2:**
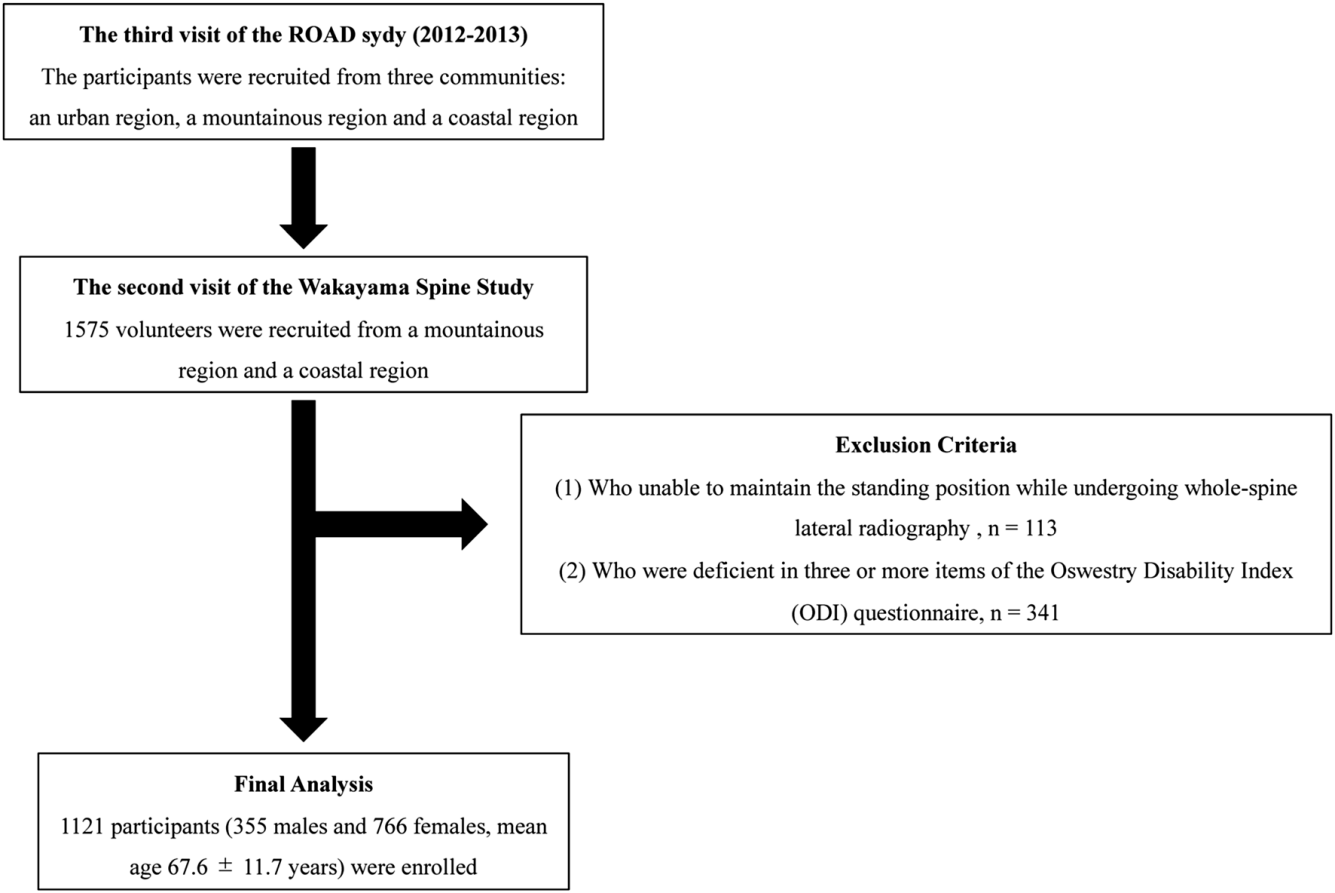
Flow diagram depicting the study enrollment strategy. Participants for the present study were recruited from among the residents of the Wakayama mountainous and coastal regions who attended the 2012–2013 visit for clinical evaluation, as a part of the Research on Osteoarthritis/osteoporosis Against Disability (ROAD) Study.

Overall, 1575 individuals (513 men and 1062 women) participated in the third visit of the ROAD study. Of the 1575 participants, 113 who could maintain the standing position while undergoing whole-spine lateral radiography were excluded. In addition, we excluded 341 patients who lacked data on three or more items of the ODI questionnaire^25^ following previous studies^26^. Finally, 1121 participants (355 men and 766 women) met the inclusion criteria (Fig. 2). Demographic measurements included height (m), weight (kg), and BMI [weight (kg)/height × 2 (m^2^)].

### Radiographic assessment

Standing lateral radiographs of the whole spine were obtained for each participant by licensed radiography technicians using a 40-inch film. Films were positioned so that the bones from C2 to the proximal femur were in the range. Each radiograph was aligned such that the film edge was the reference for vertical alignment. As described previously^27^, participants were instructed to stand in a comfortable position with their hips and knees fully extended. The arms were flexed with the hands resting on supports at the level of the shoulders. The following parameters were measured on lateral radiographs^28–30^: (1) PT was measured as the angle between the line connecting the midpoint of the sacral plate to the axes of the femoral heads and the vertical axis; and (2) C7-SVA was measured as the horizontal distance from the C7 plumb line originating at the middle of the C7 vertebral body to the posterior superior endplate of S1.

**Figure 3:**
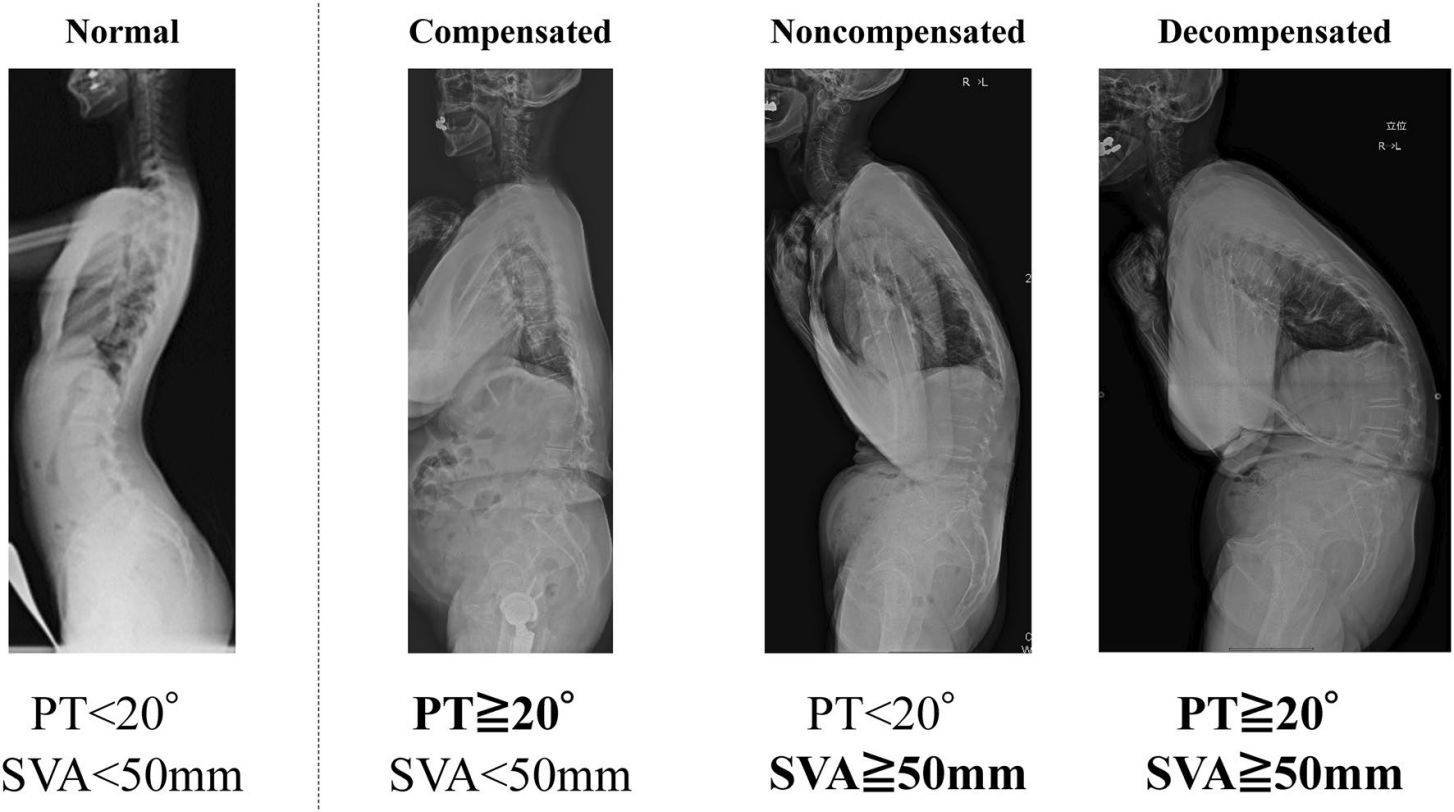
Grouping of participants. The participants were divided into the following four groups on the basis of PT and C7-SVA: normal group (PT < 20°; C7-SVA < 50 mm), compensated group (PT ≥ 20°; C7-SVA < 50 mm), non-compensated group (PT < 20°; C7-SVA ≥ 50 mm), and decompensated group (PT ≥ 20°; C7-SVA ≥ 50 mm). The latter three categories were defined as “malalignment,” and the characteristics of and factors related to each group were examined.

Participants were divided into four groups, which are original to this research (with reference to a previous study)^29^. The four groups were defined based on the PT and C7-SVA as follows: normal group (PT < 20°; C7-SVA < 50 mm), compensated group (PT ≥ 20°; C7-SVA < 50 mm), non-compensated group (PT < 20°; C7-SVA ≥ 50 mm), and decompensated group (PT ≥ 20°; C7-SVA ≥ 50 mm) (Fig. 3). The latter three categories were defined as “malalignment,” and the characteristics and factors related to each group were examined.

### Assessment of low back pain and low back pain-related disability

Experienced board-certified orthopedic surgeons asked all participants the following questions: “Have you experienced low back pain on most days during the past month, in addition to right now?” Those who answered “yes” were defined as having LBP based on previous studies^31,32^ and in the current study.

LBP-related disability was evaluated using the ODI, which calculates the degree of disability during daily activities, including personal care, lifting, walking, sitting, standing, and sleeping^25,26^. The ODI was scored from 0 to 100%, with higher scores indicating a greater degree of disability. Based on a previous report^33^, we considered patients with ODI scores of ≥ 21% as having an LBP-related disability.

### Assessment of sarcopenia

We defined sarcopenia based on the following recommended cut-off values of skeletal muscle mass according to the report of the Asian Working Group for Sarcopenia^34^: 1) age 60 or 65 years at sarcopenia diagnosis (according to country-wise definition of elderly); 2) low appendicular skeletal muscle mass (using bioimpedance analysis), 7.0 kg/m^2^ for men and 5.7 kg/m^2^ for women; 3) low handgrip strength, < 26 kg for men and < 18 kg for women; 4) low gait speed (normal gait speed ≤ 0.8 m/s).

In the present study, we considered subjects as having sarcopenia if they had a low skeletal muscle mass with low handgrip strength or low gait speed. Handgrip strength, walking speed, and muscle mass were measured as follows:

Handgrip strength was measured using a handgrip dynamometer (Toei Light Co., Ltd., Saitama, Japan). Both hands were tested, and the greater value was noted as the maximum handgrip strength. Usual walking speed was measured as the index of muscle function. The time taken (in seconds) to walk a distance of 6 m at normal walking speed in a hallway was manually assessed using a stopwatch, and the usual gait speed was calculated.

### Skeletal muscle mass

Skeletal muscle mass was measured with bioimpedance analysis^35–39^ using the Body Composition Analyzer MC-190 (Tanita Corp., Tokyo, Japan). We used the protocol described by Tanimoto et al. (as cited by Strasser et al. and Chow et al.)^40,41^, which has been validated by Nemoto et al. (as cited by Bess et al.)^42^. Appendicular skeletal muscle mass is the sum of the muscle mass of the arms and legs. Absolute appendicular skeletal muscle mass was converted to a skeletal muscle mass index by dividing the appendicular skeletal muscle mass by height in meters squared (kg/m^2^).

### Statistical analysis

Descriptive statistics were determined and are presented as mean and standard deviation or frequency and percentage, unless otherwise specified. Differences in proportions were examined using the Chi-square test. When the expected cell size was < 5, Fisher’s exact test was performed. Differences in continuous values between men and women were examined using Student’s t-test. Student’s t-test was performed after confirming that data were normally distributed using the Shapiro–Wilk normality test. Differences in proportions were examined using the Chi-square test. When significant differences were determined in the whole-group analysis, pairwise comparison was performed using Tukey’s wholly significant difference test. Risk factors for LBP, LBP-related disability (ODI% ≥ 21), and sarcopenia were evaluated using univariate and multivariate logistic regression analyses with the normal group as the reference group. When we performed multivariate logistic regression analysis, we limited the number of explanatory variables to one-tenth of the smaller number of events to ensure statistical power by referring to a previous study^45^. ORs and 95% confidence intervals as well as *p *values were calculated. Other factors related to the person were analyzed on a per person basis. In the multivariate logistic regression models, age, sex, and BMI were included in all models as they are basic cohort factors. All statistical analyses were performed using JMP Pro version 16 (SAS Inc., Cary, NC, USA). A *P *value < 0.05 was set to indicate statistical significance.

## Data Availability

The datasets generated during and/or analyzed during the current study are available from the corresponding author on reasonable request. This is not to restrict the use of our dataset but to allow the Research Ethics Committee of Wakayama Medical University and Tokyo University to understand the actual use of the dataset.
