# Publisher Correction: Pelvic compensation accompanying spinal malalignment and back pain-related factors in a general population: the Wakayama spine study

**DOI:** 10.1038/s41598-023-39895-9

**Published:** 2023-08-07

**Authors:** Shizumasa Murata, Hiroshi Hashizume, Shunji Tsutsui, Hiroyuki Oka, Masatoshi Teraguchi, Yuyu Ishomoto, Keiji Nagata, Masanari Takami, Hiroshi Iwasaki, Akihito Minamide, Yukihiro Nakagawa, Sakae Tanaka, Noriko Yoshimura, Munehito Yoshida, Hiroshi Yamada

**Affiliations:** 1 Department of Orthopaedic Surgery, Wakayama Medical University, 811-1 Kimiidera, Wakayama City, Wakayama 641-8510 Japan; 2 Division of Musculoskeletal AI System Development, Graduate School of Medicine, The University of Tokyo, Bunkyo-Ku, Tokyo, Japan; 3 Spine Center, Dokkyo Medical University Nikko Medical Center, 632 Takatoku, Nikko City, Tochigi Japan; 4 Spine Care Center, Wakayama Medical University Kihoku Hospital, 219 Myoji, Katsuragi-cho, Ito-gun, Wakayama, Japan; 5 Department of Orthopaedic Surgery, The University of Tokyo, Bunkyo-Ku, Tokyo, Japan; 6 Department of Preventive Medicine for Locomotive Organ Disorders, 22nd Century Medical and Research Center, The University of Tokyo, Bunkyoku, Tokyo, Japan; 7 Department of Orthopedic Surgery, Sumiya Orthopaedic Hospital, 337 Yoshida, Wakayama, Japan

Correction to: *Scientific Reports* 10.1038/s41598-023-39044-2, published online 22 July 2023

The original version of this Article contained an error in the order of Figures 1, 2 and 3. Figure 1 was published as Figure 3, Figure 2 was published as Figure 1 and Figure 3 was published as Figure 2.

The original Figures 1, 2 and 3 and accompanying legends appear below.

**Figure 1:**
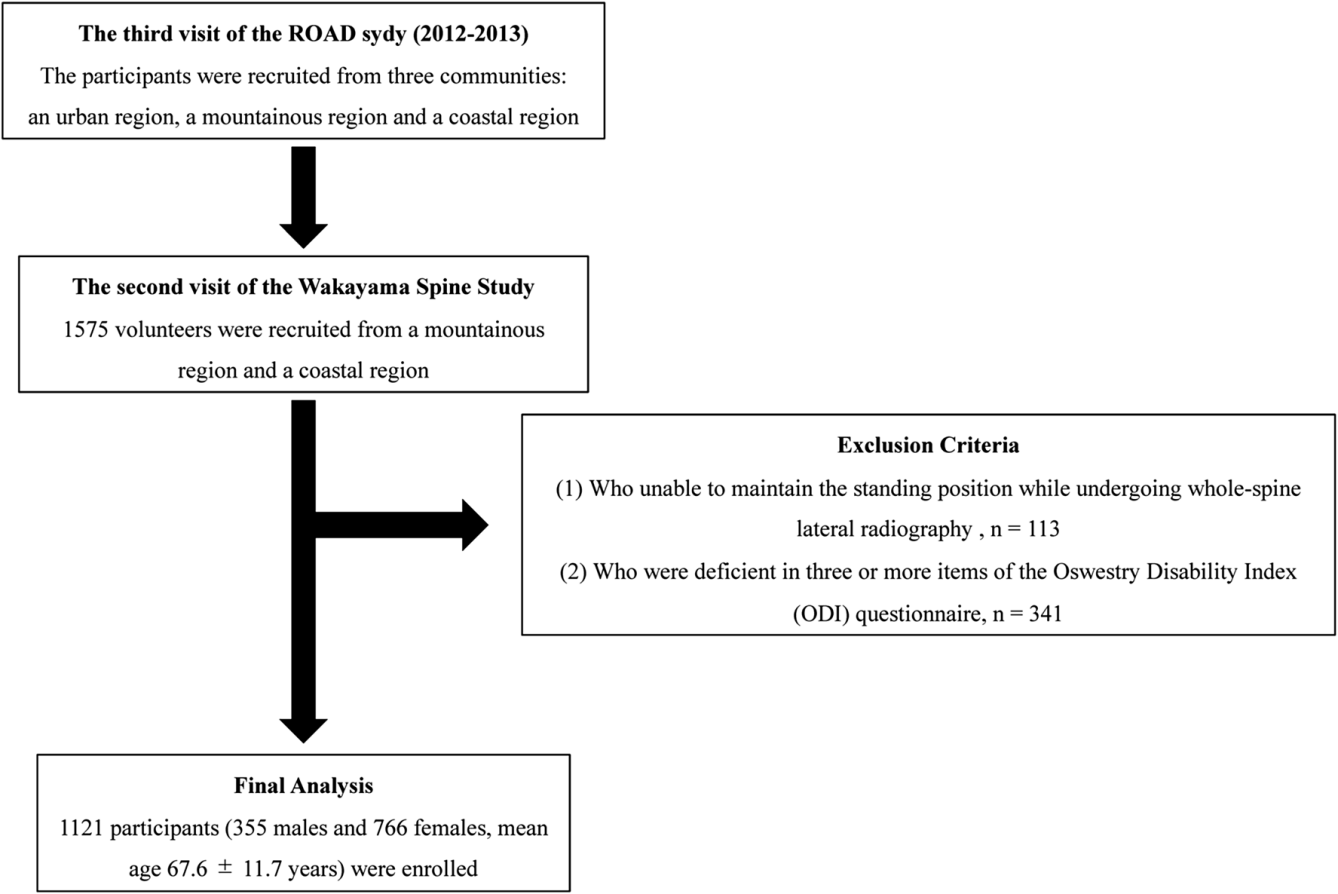
The prevalence of malalignment. The prevalence of malalignment increased with age group: < 50 years, 19%; 50 s, 29%; 60 s, 40%; 70 s, 54%; and > 80 years, 69%. In women, the prevalence of malalignment was significantly higher among those aged > 60 years (40.3% in men vs. 57.6% in women, *P* < 0.0001).

**Figure 2:**
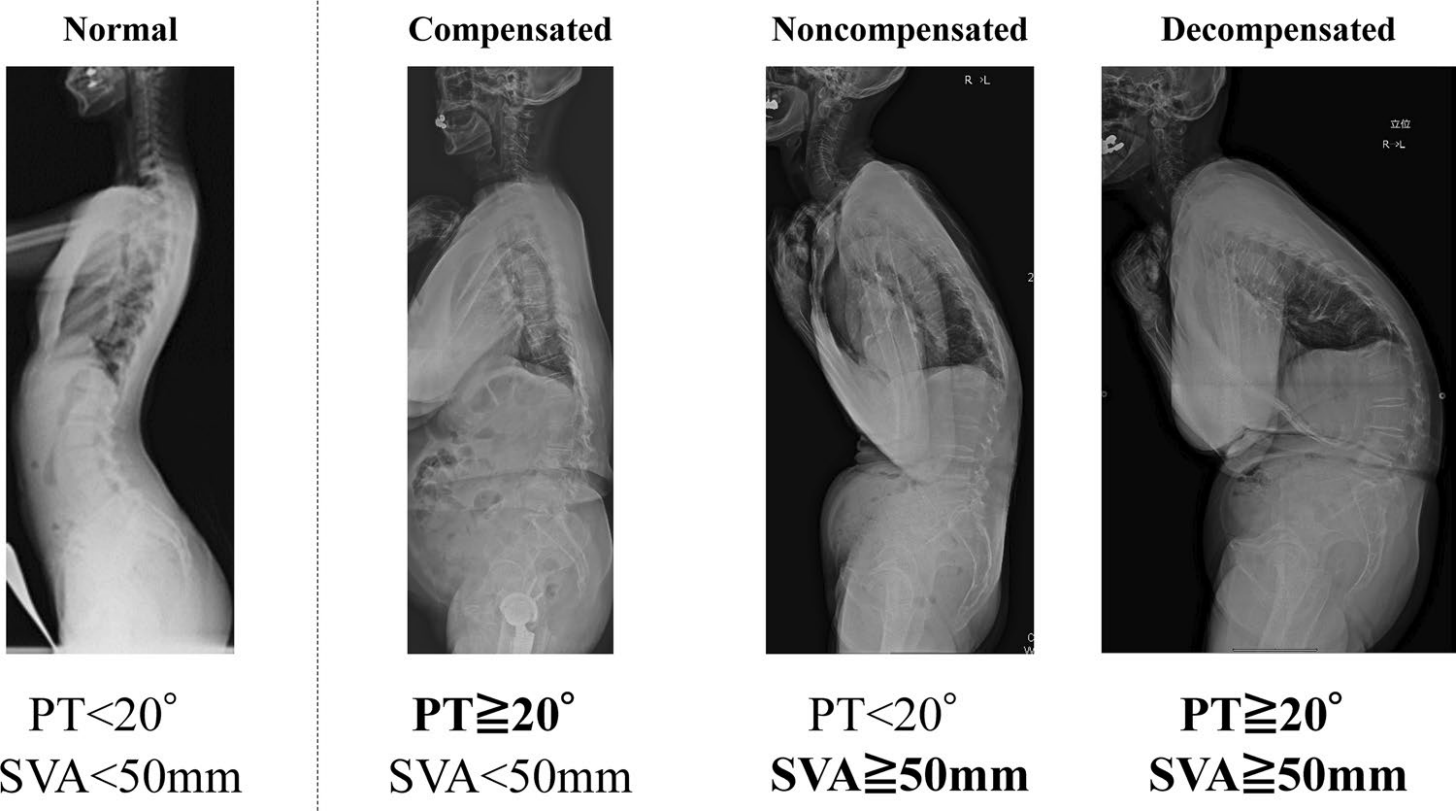
Flow diagram depicting the study enrollment strategy. Participants for the present study were recruited from among the residents of the Wakayama mountainous and coastal regions who attended the 2012–2013 visit for clinical evaluation, as a part of the Research on Osteoarthritis/osteoporosis Against Disability (ROAD) Study.

**Figure 3:**
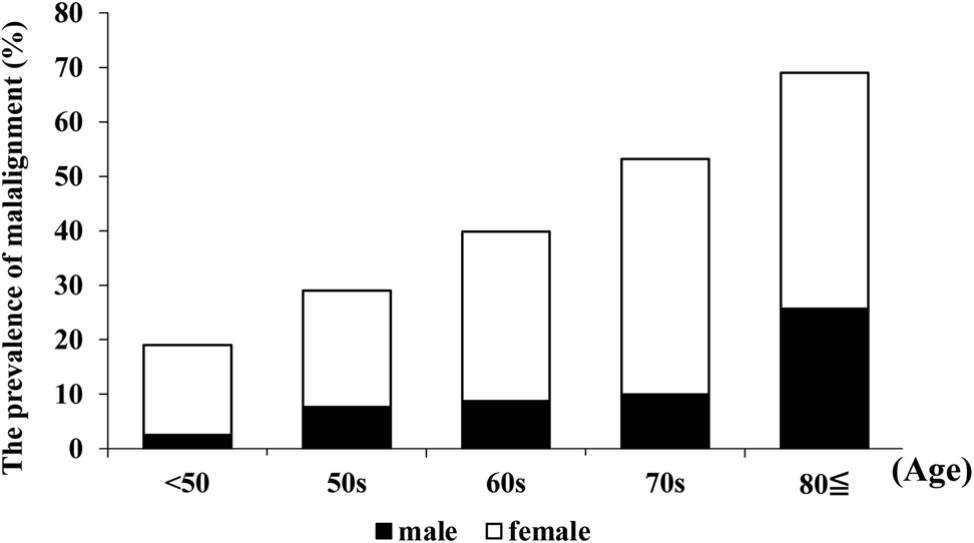
Grouping of participants. The participants were divided into the following four groups on the basis of PT and C7-SVA: normal group (PT < 20°; C7-SVA < 50 mm), compensated group (PT ≥ 20°; C7-SVA < 50 mm), non-compensated group (PT < 20°; C7-SVA ≥ 50 mm), and decompensated group (PT ≥ 20°; C7-SVA ≥ 50 mm). The latter three categories were defined as “malalignment,” and the characteristics of and factors related to each group were examined.

The original Article has been corrected.

